# Immunoglobulins response of COVID-19 patients, COVID-19 vaccine recipients, and random individuals

**DOI:** 10.1371/journal.pone.0281689

**Published:** 2023-02-14

**Authors:** Mohammad Al-Tamimi, Amjed A. Tarifi, Arwa Qaqish, Manal M. Abbas, Hadeel Albalawi, Jumanah Abu-Raideh, Muna Salameh, Ashraf I. Khasawneh

**Affiliations:** 1 Department of Basic Medical Sciences, Faculty of Medicine, The Hashemite University, Zarqa, Jordan; 2 Department of Specialized Surgery, Faculty of Medicine, The Hashemite University, Zarqa, Jordan; 3 Department of Biology and Biotechnology, Faculty of Science, The Hashemite University, Zarqa, Jordan; 4 Department of Medical Laboratory Sciences, Faculty of Allied Medical Sciences, Al-Ahliyya Amman University, Amman, Jordan; 5 Pharmacological and Diagnostic Research Lab, Al-Ahliyya Amman University, Amman, Jordan; 6 Department of Basic Medical Sciences, Faculty of Medicine, AlBalqa Applied University, Alsalt, Jordan; CCAC, UNITED STATES

## Abstract

**Background:**

The development of specific immunoglobulins to COVID-19 after natural infection or vaccination has been proposed. The efficacy and dynamics of this response are not clear yet.

**Aim:**

This study aims to analyze the immunoglobulins response among COVID-19 patients, COVID-19 vaccine recipients and random individuals.

**Methods:**

A total of 665 participants including 233 COVID-19 patients, 288 COVID-19 vaccine recipients, and 144 random individuals were investigated for anti-COVID-19 immunoglobulins (IgA, IgG, IgM).

**Results:**

Among COVID-19 patients, 22.7% had detectable IgA antibodies with a mean of 27.3±57.1 ng/ml, 29.6% had IgM antibodies with a mean of 188.4±666.0 BAU/ml, while 59.2% had IgG antibodies with a mean of 101.7±139.7 BAU/ml. Pfizer-BioNTech vaccine recipients had positive IgG in 99.3% with a mean of 515.5±1143.5 BAU/ml while 85.7% of Sinopharm vaccine recipients had positive IgG with a mean of 170.0±230.0 BAU/ml. Regarding random individuals, 54.9% had positive IgG with a mean of 164.3±214 BAU/ml. The peak IgM response in COVID-19 patients was detected early at 15–22 days, followed by IgG peak at 16–30 days, and IgA peak at 0–60 days. IgM antibodies disappeared at 61–90 days, while IgG and IgA antibodies decreased slowly after the peak and remained detectable up to 300 days. The frequency of IgG positivity among patients was significantly affected by increased age, admission department (inpatient or outpatient), symptoms, need for oxygen therapy, and increased duration between positive COVID-19 RT PCR test and serum sampling (p˂0.05). Positive correlations were noted between different types of immunoglobulins (IgG, IgM, and IgA) among patients.

**Conclusions:**

Natural infection and COIVD-19 vaccines provide IgG-mediated immunity. The class, positivity, mean, efficacy, and duration of immunoglobulins response are affected by the mechanism of immunity and host related variables. Random community individuals had detectable COVID-19 IgG at ~55%, far from reaching herd immunity levels.

## Introduction

An outbreak of the novel (new) coronavirus was first reported in December 2019 in Wuhan, Hubei Province, China. In March 2020, coronavirus disease 2019 (COVID-19), caused by severe acute respiratory syndrome coronavirus 2 (SARS-CoV-2), was declared as pandemic by the World Health Organization (WHO). With the emergence of many variants, SARS-CoV-2 spread continued leading to the greatest hardship of public health, social development, and economy in our times. As of February 10, 2022, there have been more than 400 million cases worldwide with over 5.5 million deaths [1, 2].

SARS-CoV-2 is the newest member of the Betacoronavirus family, which also includes the causative agents of SARS-CoV and Middle East respiratory syndrome virus (MERS) [3]. Four essential proteins are encoded by the 30 Kb +ve strand RNA genome of the virus: S: spike, N: nucleocapsid, M: membrane and E: envelope, in addition to 15 non-structural proteins (Nsp1-10 and Nsp12-16), and 8 accessory proteins [4, 5].

The S protein is of special importance as it mediates attachment and subsequent viral entry into the target cell. The receptor binding domain (RBD) of the S1 subunit mediates attachment to the membrane of a host cell through binding to angiotensin-converting enzyme 2 (ACE2). The S2 subunit mediates membrane fusion allowing viral entry [6, 7]. Hence, S has been the main target for the development of vaccines and immuno-therapeutics [8].

Antibodies (Abs) specific to SARS-CoV-2 have been extensively studied in the course of natural infection as well as vaccination. Immunoglobulin A (IgA), immunoglobulin M (IgM), and immunoglobulin G (IgG) against S and N proteins of SARS-CoV-2 evolve rapidly within 1 to 2 weeks after symptoms onset in the sera of COVID-19 patients [9–12]. Specific COVID-19 IgG antibodies continue to rise months after infection and would possibly remain active for more than a year [13]. Disease severity proved to reflect on titers and kinetics of the COVID-19 Ab response. It has been repeatedly reported that asymptomatic and mildly symptomatic cases have markedly lower serum Ab titers that wane more rapidly compared to symptomatic patients [14, 15]. On the other hand, the potential development of acute respiratory distress syndrome (ARDS), proved to correlate very strongly with higher Ab titers, especially against the N protein [16]. Interestingly, it was reported that deceased patients show slower appearance of Abs in their sera although titers reach higher levels later in disease progress [17, 18].

Antigen specific IgA response appears to be stronger and more persistent than the IgM response [19]. IgA was found to be predominant in the early phase of SARS-CoV-2 infection in sera and it remained for a longer period in mucosal surfaces of patients [20]. IgA from sera, saliva and bronchoalveolar lavages of patients proved to be more potent in viral neutralization compared to IgG. Interestingly, IgA dimers at the mucosal surfaces showed to be 15 times more potent in virus neutralization than serum IgA monomers [21]. This suggests that IgA Abs might have an important role in preventing infection, transmission and worsening of symptoms.

Specific immunity after COVID-19 vaccination has been documented. Most reports revealed that IgG levels were significantly higher in mRNA vaccinated groups compared to naturally infected patients. On the other hand, IgG levels in vaccinees who took inactivated virus vaccines were more similar to those of natural infection [22–26].

COVID-19 mRNA vaccines also elicit antigen-specific IgA levels and kinetics similar to naturally infected patients [27]. However, only one study reported the IgA response in inactivated virus vaccinees. This study stated that, in contrast to mRNA based Comirnaty, the inactivated virus CoronaVac did not elicit detectable IgA in the nasal mucosa, but both showed detectable serum IgA [28].

The efficacy, kinetics, and protection after natural and vaccine-induced COVID-19 immunity are not fully understood. Specific antibodies play essential role in COVID-19 protection through neutralization and clearance effects. In this study we aimed to investigate the frequency, titer, efficacy, and kinetics of immunoglobulins response (IgG, IgM, and IgA) of COVID-19 patients, COVID-19 vaccine recipients and random individuals.

## Materials and methods

### Study design

The sampled population included randomized Jordanian individuals who have been infected with COVID-19 confirmed by RT-PCR from May 2020 to August 2021. Participants were recruited at Prince Hamza Hospital (PHH) inpatient or outpatient departments. Demographic and clinical data were collected after obtaining a voluntary consent. Each participant then provided a serum sample for immunological studies of COVID-19 antibodies including IgA, IgM, and IgG. The duration between positive RT-PCR test and serum samples collection was calculated as days.

The details of COVID-19 vaccinated individuals were previously reported [22, 29]. Briefly, Pfizer-BioNTech vaccine recipients (n = 141), and Sinopharm vaccine recipients (n = 147) had serum collected 2 weeks after administration of the second dose. Serum samples were assayed for COVID-19 IgG and IgM antibodies [22]. Finally, 144 random individuals were recruited to determine community prevalence of COVID-19 immunoglobulins. Serum samples referred to PHH labs for non-COVID related studies were collected in the period between 1 September 2021 to 1 October 2021. These samples would presumably include symptomatic and asymptomatic COVID-19 infected individuals, vaccinated individuals, and naïve individuals. Only demographic data was collected for this group without clinical or risk factors information. Serum COVID-19 IgG and IgA were then investigated. The study was approved by the institutional review board (IRB) committee at The Hashemite University on 7 March 2020 (No: 1∕5∕2019∕2020) and PHH IRB committee on 15 March 2020 (No: 1/631). All enrolled participants gave a written informed consent prior to participation in this study.

### Anti-COVID-19 immunoglobulin’s measurement

Vitek Immuno Diagnostic Assay Systems (VIDAS^®^, Biomerieux inc., Hazelwood, MO, USA) for SARS-COV-2 are automated qualitative assays that were used for the detection of IgG or IgM Abs specific for SARS-CoV-2 in human serum or plasma (lithium heparin) by utilizing Enzyme Linked Fluorescent Assay (ELFA) technique. These assays combine a two-step sandwich enzyme immunoassay method with a final fluorescence detection (ELFA). IgG Abs are specifically detected by anti-human IgG, which is labeled with alkaline phosphatase, while IgM Abs are specifically detected similarly by anti-human IgM, also labelled with alkaline phosphatase. The intensity of fluorescence is directly proportional to the level of antibody in the studied sample. An index is calculated as a ratio between the relative fluorescence value (RFV) measured in the sample and the RFV obtained for the calibrator, which is humanized recombinant anti-SARS CoV-2 IgG or IgM. The results were first interpreted as positive (index ≥1) or negative (index <1), before being converted into binding antibody units per milliliter (BAU/ml) that correlate with the WHO standard.

For quantitative determination of human anti-SARS-CoV-2 S1 protein (IgA class antibodies) in serum or plasma samples, an ELISA test was used according to manufacturer instructions (MyBioSource Inc, San Diego, CA, USA). Briefly, 96-well plates were coated with SARS-CoV-2 S1 protein. After washing, captured IgA was detected by anti-human IgA monoclonal antibodies conjugated with horse radish peroxidase (HRP). After another washing step, the chromogenic substrate 3,3’,5,5’-tetramethylbenzidine (TMB) was added and the color reaction was stopped by 2M H2SO4. Finally, the absorbance of each well was measured at 450 nm, and readings were converted to concentration (ng/ml) by blotting against a standard curve. All COVID-19 antibody detection assays used in this study are Conformite Europeenne (CE) approved and had a sensitivity and specificity rates over 90%.

### Statistical analysis

For statistical analysis, we used the Statistical Package for the Social Sciences (SPSS) version 24.0 (Chicago, IL, USA). After applying descriptive statistics, data were presented as numbers (percent) for categorical variables and mean ± standard deviation (SD) for numeric variables. Chi-squared test and fisher’s exact test were used to compare categorical variables. Correlations between IgG, IgM, and IgA titers were tested using Bivariate Pearson’s correlation test. P-value <0.05 was considered statistically significant.

## Results

### Demographic and clinical characteristics of study populations

A total of 665 participants were recruited voluntarily in the study including COVID-19 confirmed patients (n = 233) with documented positive COVID-19 RT-PCR test, COVID-19 vaccine recipients (n = 288), and random samples from PHH (n = 144). Regarding COVID-19 patients, the mean age ± SD was 39.3 ± 14.9 years, range from 2 to 80 years, with most patients in the age range of 20–41 years (48.1%). Males accounted for 52.8% while females accounted for 47.2% of participants. 65.7% of patients were outpatients with no or mild symptoms while 34.3% were admitted to hospital due to moderate to severe illness. Only 1.7% of participants were infected twice with COVID-19 and 1.7% were pregnant at time of infection. Most patients have documented COVID-19 related symptoms (64.4%) while 28.3% were asymptomatic. Furthermore, only 10.7% of patients did need oxygen therapy. The duration between infection (positive RT-PCR) and serum sampling (Mean ± SD) was 82.3 ± 72.9 days (Table 1) (S1 Table). A total of 144 random patients were recruited over 30 days with a mean age of 48.1 ± 20.5 years including 67 participants (46.5%) aged 61 to 80 years. Males were 86 (59.7%), 35 (24.3%) were outpatients, and 109 (75.7%) were inpatients (Table 1). The demographic, side effects, and clinical data related to vaccine recipients were reported in detail (Table 1) [22, 29].

**Table 1:** Demographic and clinical data of COVID-19 patients (n = 233), COVID-19 vaccine recipients (n = 288), and random patients from PHH (n = 144).

	Variable	COVID-19 confirmed Number (%)	COVID-19 vaccine recipients Number (%)	Random patients PHH Number (%)
**Age (Years)**	0–20	19 (8.2)	0 (0)	13 (9.0)
	21–40	112 (48.1)	43 (14.9)	31 (21.5)
	41–60	70 (30.0)	94 (32.6)	33 (22.9)
	61–80	20 (8.6)	151 (52.4)	67 (46.5)
	NA	12 (5.2)		
**Gender**	Male	123 (52.8)	189 (65.6)	86 (59.7)
	Female	110 (47.2)	99 (34.4)	58 (40.3)
**Patients type**	Outpatient	153 (65.7)	288 (100)	35 (24.3)
	Inpatients	80 (34.3)	0 (0)	109 (75.7)
**COVID-19 RT-PCR**	Positive	233 (100)	8 (2.8)	NA
	Negative	0 (0)	280 (97.2)	
**Number of COVID-19 Infections**	One	229 (98.3)	8 (2.8)	NA
	Two	4 (1.7)	0 (0)	
**Pregnancy**	Yes	4 (1.7)	0 (0)	NA
	No	129 (98.3)	288 (100)	
**COVID-19 Vaccine**	Yes	0 (0)	288 (100)	NA
	No	233 (100)	0 (0)	
**Duration between infection/vaccine and sampling (days)**	0–100	112 (48.1)	288 (100)	NA
	101–200	97 (41.6)	0 (0)	
	˃200	9 (3.9)	0 (0)	
	NA	15 (6.4)		
**Symptoms**	Yes	150 (64.4)	0 (0)	NA
	No	66 (28.3)	288 (100)	
	NA	17 (7.3)		
**Need for Oxygen**	Yes	25 (10.7)	0 (0)	NA
	No	190 (81.5)	288 (100)	
	NA	18 (7.7)		

NA: not available.

### Immunoglobulins response of COVID-19 patients, COVID-19 vaccine recipients, and random individuals

Overall, only 22.7% of COVID-19 infected patients had detectable COVID-19 IgA antibodies with a mean of 27.3 ± 57.1 ng/ml, 29.6% had COVID-19 IgM antibodies with a mean of 188.4 ± 666.0 BAU/ml, while 59.2% had positive COVID-19 IgG antibodies with a mean of 101.7 ± 139.7 BAU/ml (Table 2) (S1 Table). Regarding random samples, the percentage of individuals with positive COVID-19 IgG was 54.9% (79/144), with a mean of 164.3 ± 214 BAU/ml (Table 2). Finally, Pfizer-BioNTech vaccine recipients had positive COVID-19 IgG in 99.3% with a mean of 515.5 ± 1143.5 BAU/ml while 85.7% of Sinopharm vaccine recipients had positive IgG with a mean of 170.0 ± 230.0 BAU/ml (Table 2).

**Table 2:** COVID-19 immunoglobulins response for COVID-19 confirmed patients (n = 233), Pfizer-BioNTech vaccine recipients (n = 141), Sinopharm vaccine recipients (n = 147), and random patients (n = 144).

	Confirmed COVID-19	Random patients	Sinopharm vaccine	Pfizer-BioNTech vaccine
**IgG positive Frequency (%)**	138 (59.2)	79 (54.9)	126 (85.7)	140 (99.3)
**IgG Titer BAU/ml Mean (SD)**	101.7 (139.7)	164.3 (214.0)	170.0 (230.0)	515.5 (1143.5)
**IgM positive Frequency (%)**	69 (29.6)	NA	21 (26.9)	19 (20.4)
**IgM Titer BAU/ml Mean (SD)**	188.4 (666.0)	NA	26.1 (45.6)	17 (29.9)
**IgA positive Frequency (%)**	53 (22.7)	26 (18.0)	30 (20.4)	17 (12.0)
**IgA Titer ng/ml Mean (SD)**	27.3 (57.1)	4.28 (4.13)	3.56 (4.41)	2.62 (3.08)

NA: not available.

**Fig 1:**
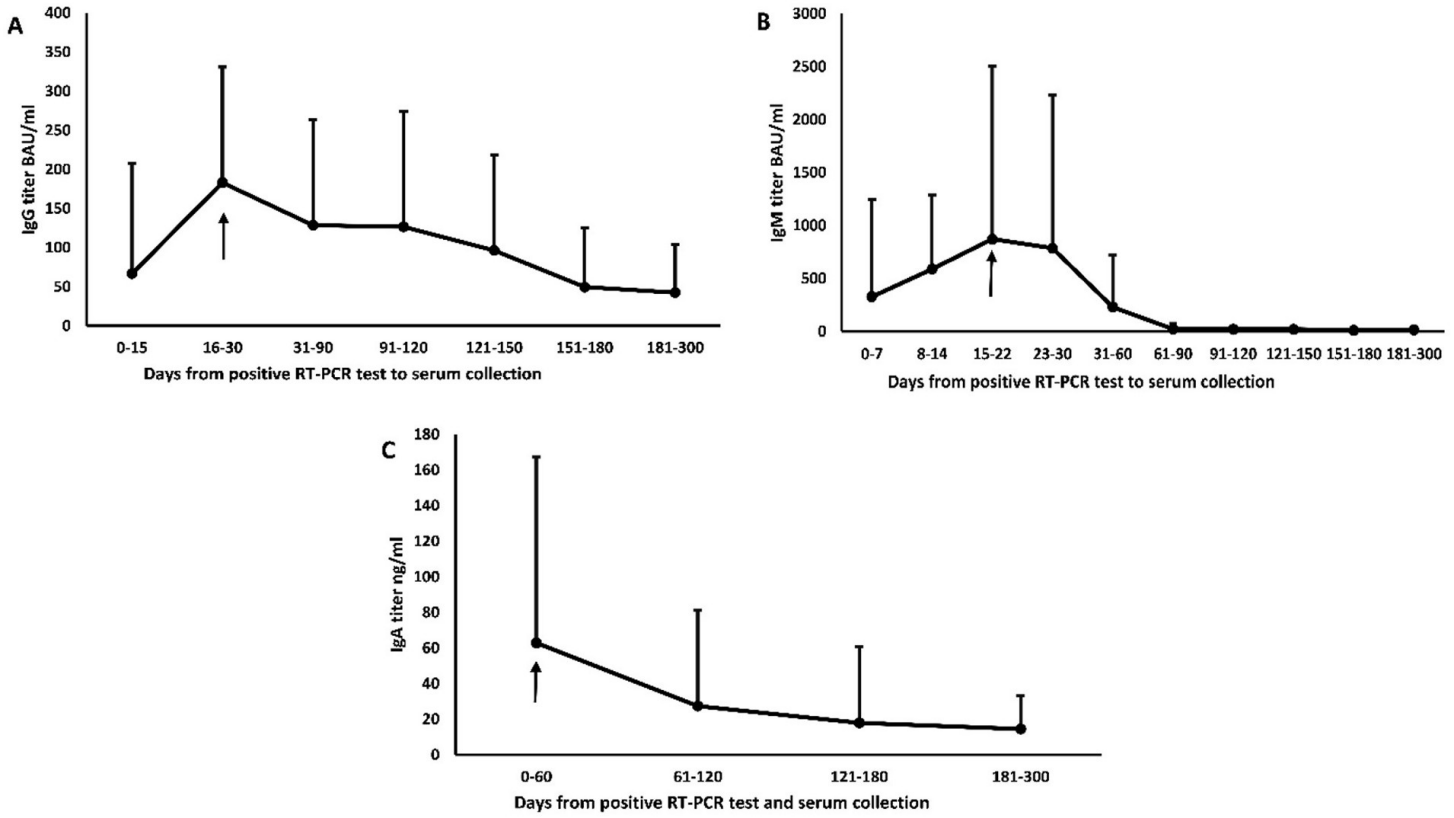
Dynamic of immunoglobulins response for COVID-19 confirmed patients (mean ± SD, n = 233). (A) IgG, (B) IgM, and (C) IgA. The black arrow indicates the peak response.

The dynamics of immunoglobulins response of COVID-19 patients were studied to determine the peak response of each immunoglobulin in COVID-19 patients (Fig 1). Th peak IgG response in COVID-19 patients was at 16–30 days (183.1 ± 147.9 BAU/ml) (Fig 1A). The peak IgM response in COVID-19 patients was early at 15–22 days (872.3 ± 1634.2 BAU/ml) (Fig 1B). The peak IgA response in COVID-19 patients was at 0–60 days (62.9 ± 104.3 ng/ml) (Fig 1C). IgM antibodies almost disappeared at 61–90 days in all patients, while IgG and IgA antibodies started to decrease slowly after the peak and were still detectable up to 300 days (Fig 1) (S2 Table).

### Factors affecting immunoglobulins positive response among COVID-19 patients

The frequency of IgG positivity was significantly affected by increased age (p = 0.02), outpatients (p < 0.001), symptomatic patients (p < 0.001), patients who needed oxygen therapy (p = 0.01), and increased duration between positive COVID-19 RT-PCR test and serum sampling (Table 3). Furthermore, the frequency of IgM positivity was significantly increased in inpatients (p = 0.02), patients who needed oxygen (p = 0.004), and shorter duration between positive COVID-19 RT-PCR test and serum sampling (p = 0.02) (Table 3). There were no factors that had any significant effect on the frequency of IgA positivity among COVID-19 patients (Table 3) (S1 Table).

**Table 3:** Association of COVID-19 IgG, IgM, and IgA among COVID-19 patients with demographic and clinical data (n = 233).

		COVID-19 IgG	p-value	COVID-19 IgM	p-value	COVID-19 IgA	p-value
**Age**	0–20	6 (35.3)	0.02	5 (26.3)	0.12	2 (66.7)	0.49
	21–40	70 (64.2)		27 (24.1)		28 (33)	
	41–60	42 (64.6)		26 (37.1)		16 (43.2)	
	61–80	11 (57.9)		9 (45)		4 (33.3)	
**Gender**	Male	67 (58.8)	0.28	36 (29.3)	0.86	30 (41.1)	0.30
	Female	71 (65.7)		33 (30.3)		25 (32.9)	
**Patients type**	Outpatient	109 (71.2)	<0.001	38 (25)	0.02		
	Inpatients	29 (42.0)		31 (38.8)			
**Number of infections**	One	134 (63.3)	0.11	68 (29.8)	0.83	53 (36.6)	0.58
	Two	4 (100.0)		1 (25)		2 (100)	
**Pregnancy**	Yes	2 (50.0)	0.61	0 (0.0)	0.18	1 (33.3)	0.89
	No	136 (62.4)		69 (30.3)		54 (37)	
**Symptoms**	Yes	109 (74.1)	<0.001	47 (31.3)	0.88	47 (38.5)	0.59
	No	23 (39.7)		20 (30.3)		3 (30)	
**Need for oxygen**	Yes	21 (87.5)	0.01	14 (56)	0.004	8 (53.3)	0.19
	No	110 (61.1)		53 (27.9)		42 (35.9)	
**Duration (days)**	0–100	40 (48.5)	<0.001	42 (37.5)	0.02	11 (35.5)	0.51
	101–200	76 (78.4)		23 (23.7)		34 (35.4)	
	201–300	5 (55.6)		2 (22.2)		4 (57.1)	

### Correlations between immunoglobulins among COVID-19 patients

IgG titer correlated positively with IgM titer and IgA concentration (p < 0.001) among COVID-19 patients. In addition, IgG positive results were associated significantly with positive IgM and positive IgA results (p < 0.001). Furthermore, IgM titer correlated positively with IgA concentration and was associated with positive results (p < 0.001) (Table 4) (S1 Table).

**Table 4:** Correlation between different types of immunoglobulins among COVID-19 patients.

		**IgM titer BAU/ml**	**IgA concentration ng/ml**
**IgG titer BAU/ml**	Pearson correlation (r)	0.346	0.302
	Significance (p-value)	<0.001	<0.001
		**IgM Positive**	**IgA positive**
**IgG positive**	Frequency (%)	56 (82.4)	50 (90.9)
	Significance (p-value)	<0.001	<0.001

## Discussion

Magnitude and kinetics of humoral immunity in response to SARS-CoV-2 infection give insights into measures of immune protection against natural infection as well as measures of vaccine efficacies. It has been repeatedly reported that most B-cell epitopes identified have been found in the S1 C-terminal domain and S2 [30–32]. In addition, approximately most neutralizing Abs are induced by the RBD (amino acids 306–527) and S2 [33, 34]. Since the S2 protein is more conserved within other corona-viruses, S1 (specifically the RBD) remains the most valuable tool for specific Ab identification [34–44]. Hence, in this study, we measured the presence of S1 specific IgM, IgG and IgA in sera from naturally infected COVID-19 patients, COVID-19 naïve Pfizer and Sinopharm vaccinated personnel, and a group of random individuals.

The positivity and levels of anti-S IgG were found to negatively correlate with age and positively with symptom severity, which is consistent with previous studies [45, 46]. Increased symptoms severity, late Ab response, and elevated viral loads could stand behind the higher Ab concentration in sera of older patients compared to younger ones, especially at longer times (6 months) post infection. Previous studies reported that anti-S Ab titers increased with symptom severity [45, 46]. A significant positive correlation was found between anti-S IgG and patients department. Naturally infected unvaccinated outpatients showed higher Ab responses compared to inpatients. This could be due to variations in blood collection time. Inpatients samples were collected during hospitalization within the first 2 weeks after the onset of COVID-19 symptoms, whereas outpatients donated their samples up to 10 months after.

In the context of vaccination, we found that anti-S IgG levels detected 2 weeks post-vaccination were highest among COVID-19 naïve Pfizer-BioNTech vaccinated individuals consistent with previous investigations, as Pfizer-BioNTech was repeatedly reported to be more effective in immune protection against COVID-19 compared to Sinopharm and natural infection [22–26, 39–41, 47].

IgM is thought to play an important role in protective immunity against COVID-19, as a strong association between declining anti-S IgM levels and declining neutralizing Ab responses was observed [48–50]. Here, we found that the IgM response in naturally infected COVID-19 patients was higher compared to vaccinated individuals in terms of positivity and titers, which is consistent with other studies [42, 50]. Interestingly, Ruggiero et al. [50] reported that COVID-19 naïve individuals vaccinated with Pfizer’s mRNA vaccine showed unconventional patterns of anti-S IgM responses depicted by either absence of IgM, development of IgM after IgG, or simultaneous presence of IgM and IgG. It is hard to speculate the reason behind vaccine induced unconventional IgM responses in potentially COVID-19 naïve vaccinated personnel. One reason for the total absence of IgM two weeks post full vaccination could be the lack of IgM memory response from a pre-existing immunity to cross-reactive human coronaviruses, a previous primary immune response against an asymptomatic infection with the virus, or against first booster of vaccination with expedited IgM decay. Another probable reason could be the adjuvant effect of the lipid components of the vaccine in driving early and extensive IgG class-switching because of the reported Th1-polarized responses [51]. The persistence of virus-specific IgM responses in vaccinees could refer to the persistence of non-class-switched IgM+ memory B cells [52].

SARS-CoV-2 S IgA elicited by natural infection mediates viral neutralization and is likely an important component of natural immunity [27, 28]. IgA responses to COVID-19 vaccines have been investigated, especially against mRNA-based vaccines. Chan et. al. reported that COVID-19 mRNA vaccination evokes S specific IgA with similar kinetics compared to S specific IgG but it declines more rapidly in sera of vaccinees following both the 1st and 2nd doses [28].

It has been reported that both mRNA based Comirnaty and inactivated virus CoronaVac induce plasma SARS-CoV-2 S1-specific IgA. However, Comirnaty, but not CoronaVac, was also able to induce S1-specific IgA in the nasal mucosa [28]. mRNA based vaccines also evoked the secretion of anti-S IgA in women milk [53] as well as saliva of vaccinees [54]. Although the intramuscular route of vaccination does not induce mucosal immunity [55], there has been evidence that lipid nanoparticles, such as those harboring the mRNA-based vaccines can be detected in distal tissues, including the lung [56]. Our findings show that IgA levels are higher in naturally infected COVID-19 patients compared to Pfizer and Sinopharm vaccinees and random individuals, indicating that IgA response is more prominent due to natural infections consistent with previous investigations [27, 28].

We find it hard to draw conclusions on our random individual group, as it could represent a variety of infection/vaccination settings including naturally infected-unvaccinated personnel, COVID-19 naïve vaccinees with different types of vaccines (Pfizer, Sinopharm or AstraZeneca) and partial or full vaccination, and vaccinees with previous or concurrent infection. However, the Ab response of this group with ~55% anti-S IgG is still far from reaching herd immunity levels (65–95%) among the Jordanian population [57].

Initial studies during the first wave of COVID-19 spread, before the introduction of vaccines and before probable reinfections, reflected a realistic pattern of immunoglobulins response to SARS-CoV-2 infection. In general, IgM peak response was at 2–4 weeks and became undetectable 3 months post symptoms onset, IgG and IgA antibodies usually follow and peaked at around 30–60 days then decrease slowly with levels still detectable 9 months or later [58–60]. Despite heterogeneity of the sample population, severe COVID-19 cases were always associated with higher Ab production and neutralization titers [60]. A large cohort study on mRNA-based vaccine recipients reported that the IgG response evoked against vaccination for COVID-19 peaked 15 days post second dose and declined over time through six months post vaccination [61]. Despite waning antibody titers over time after vaccination, no cases of severe COVID-19 were detected among participants in another study [62]. The kinetics of COVID-19 immunoglobulins reported in this study are in concordance with previous studies [36–44, 58–62].

Despite the importance of neutralizing Abs in protection against SARS-CoV-2 infection, the other arm of adaptive immunity, namely T cells, has proved to be important for immune protection against COVID-19. Virus specific cytotoxic CD8+T cell response was detected early within 7 days of symptom onset and peaked after 14 days. This proved to correlate with effective viral clearance and milder symptoms. On the other hand, T cell responses were found to be severely impaired in severe and critical cases of COVID-19. This impairment was found to be associated with intense T cell activation and lymphopenia [60–62].

## Conclusions

Specific SARS-CoV-2 anti-S antibodies were detectable in naturally infected, vaccine recipients and random individuals. The class, levels, positivity rate, dynamics and duration of immunoglobulins response varied widely, which reflect immunogenicity and boosting effect at one end and host immune state at the other end. This study highlights the complexity and diversity of factors contributing to COVID-19 immunoglobulins response among community.

## Supporting information

S1 Table Raw data of COVID-19 confirmed patients. Raw data of study population including demographic data, clinical data, and immunoglobins response (IgG, IgM, and IgA). These data were used to generate Tables 1–4.
(XLS)

S2 Table Raw data of immunoglobulins response of COVID-19 confirmed patients over time. Immunoglobulins titers mean ± SD over time in days was provided and used to generate Fig 1.
(XLSX)
